# Phenotypic and genomic characterization of tigecycline-insensitive *Klebsiella pneumoniae* strains and validation of highly virulent MDR strains

**DOI:** 10.1128/spectrum.03228-25

**Published:** 2026-04-08

**Authors:** Lu Liu, Caiting Niu, Rongmao Zong, Baosheng Duan, Xiaodan Li, Fang Jia

**Affiliations:** 1 Research Center of Molecular Biology, College of Basic Medicine, Inner Mongolia Medical University 66287 , Hohhot, China; 2 Ordos Central Hospital 688286 , Ordos, China; 3 First Clinical Medical College, Inner Mongolia Medical University, Hohhot, China; University of Minnesota Twin Cities, Minneapolis, Minnesota, USA

**Keywords:** *Klebsiella pneumoniae*, tigecycline, multidrug resistance, highly virulent strain, whole-genome sequencing

## Abstract

Tigecycline is often used as a last-resort antibiotic to combat multidrug-resistant *Klebsiella pneumoniae* (MDR-KP). However, reports of tigecycline resistance are increasing. Thirty-two tigecycline-insensitive *K. pneumoniae* (TIKP) strains were detected among 152 MDR-KP isolates obtained from a tertiary hospital in China (21%). Whole-genome sequencing (Illumina NovaSeq 6000) revealed that these 32 TIKP isolates carried 119–127 virulence genes, 38–63 resistance genes, and 21 distinct sequence types (STs), including a novel ST (8597), together with 102,632 single-nucleotide polymorphisms. The predominant plasmid types included IncFIB(K) (10/32), IncFII(K) (11/32), IncR (11/32), RepB (13/32), and IncHI1B(pNDM-MAR) (12/32). The biofilm formation and serum resistance of the four strains were tested with a *Galleria mellonella* infection assay and whole-genome sequencing (PacBio platform). Among these strains, strain 10 showed strong biofilm formation and the strongest serum resistance. Compared with the other strains, it carried the most plasmids, and its chromosomes and plasmids harbored numerous resistance genes and contained more tigecycline resistance genes. These findings demonstrate the complex genetic features and potential transmission mechanisms of TIKP isolates.

**IMPORTANCE:**

Our study revealed a high proportion (21%) of tigecycline-insensitive *K. pneumoniae* (TIKP) strains. We further characterized these TIKP isolates phenotypically and genomically, confirming the presence of highly virulent multidrug-resistant (MDR) strains. We discovered a novel allele (phoE 813) and a novel ST (8597). Our findings are significant because phenotypic analysis of TIKP allows clear determination of the level of tigecycline resistance (TRKP), which aids in optimizing antibiotic therapy and supports more rational clinical drug use. In addition, the plasmid-mediated resistance genes (e.g., the concurrent presence of both *mcr-8* and *tmexCD–toprJ* in strain 10 in this study) may spread through the environmental–animal–human transmission chain. These results provide valuable insights for developing strategies to monitor and control the spread of TIKP.

## INTRODUCTION

*Klebsiella pneumoniae* (KP) is a species of gram-negative bacteria commonly found in the human upper respiratory tract and intestines. The nasopharynx has a rate of approximately 19% colonization. In addition, older people have higher rates of oropharyngeal colonization. An epidemiological analysis of intestinal colonization in healthy adults in six Asian countries revealed that the total colonization rate of KP was more than 60% (1). KP is also an important opportunistic pathogen in the family Enterobacteriaceae and an iatrogenic infectious bacterium with strong pathogenicity in humans and high mortality rates (2). KP is highly resistant to the antibiotics commonly used in clinical practice, such as third-generation cephalosporins and aminoglycosides. The overuse of carbapenems, which are often utilized against multidrug-resistant (MDR) infections, has led to the emergence of carbapenem-resistant KP (CRKP). Patients in the ICU often have higher carriage rates, and the rate of CRKP colonization in patients in a Chinese ICU is 20.8%–28% (1). More alarmingly, carbapenem-resistant hypervirulent KP (CR-hvKP) strains have also emerged (3). HvKP is best described as a virulent pathogen, and features that are highly suggestive of hvKP infection include its ability to infect healthy individuals of any age and the propensity of infected patients to present with multiple sites of infection and/or develop subsequent metastatic spread (4). The dwindling availability of effective antibiotics against these strains poses a significant challenge for the treatment of hvKP infections.

Tigecycline, a glycylcycline antibiotic, binds reversibly to the bacterial 30S ribosomal subunit to inhibit protein translation. Chemical modifications have enhanced the binding affinity of tigecycline, allowing it to circumvent the mechanism by which tetracyclines resist ribosomal-protective proteins (5). Consequently, tigecycline has been regarded as the “last line” of treatment for infections caused by MDR gram-negative bacteria (including MDR-KP) (6). However, over the past decade, with the increased clinical use of tigecycline, reports of tigecycline resistance have arisen (7). The primary mechanisms of this resistance are mediated by the resistance–nodulation–division (RND) family, major facilitator superfamily, multidrug and toxic compound extrusion family, ATP-binding cassette superfamily, and Tet family (8). These efflux mechanisms are diverse and complex, and novel Tet(X) variants continue to emerge. Therefore, the genomic surveillance of tigecycline resistance genes and the emergence of highly virulent MDR strains are crucial.

We hypothesize that the proportion of tigecycline-insensitive *K. pneumoniae* (TIKP) strains is high, that highly virulent strains are present, and that these strains have particularly complex resistance characteristics in MDR-KP. Therefore, 32 TIKP strains were isolated from 152 MDR strains between January 2021 and December 2023 at a tertiary hospital in Inner Mongolia, China. We analyzed the genotypic, phenotypic, virulence, and phylogenetic characteristics of these TIKP strains to monitor the changing characteristics of their resistance gene and virulence gene profiles and to elucidate the characteristics of these highly virulent MDR tigecycline-resistant KP (TRKP) strains. Our findings reveal not only the types and quantities of drug resistance genes and virulence genes, ST sequences, and plasmid types, but also the strong biofilm formation ability, greater serum resistance, and high virulence characteristics of TRKP strains.

## MATERIALS AND METHODS

### Bacterial strains

In this study, 724 KP strains were isolated from clinical samples from January 2021 to December 2023 at a tertiary hospital in Ordos city, Inner Mongolia, China. Strains from the same patient and the same site, as well as those with incomplete clinical data or contamination, were excluded. The clinical information of all the strains was retrieved and sorted through the Hospital Information System. A total of 152 MDR strains (resistant to ≥3 antimicrobial classes) were identified and confirmed in this study with an automated microbial identification system (VITEK-2 Compact). Quality control (QC) strains of *Klebsiella pneumoniae* (ATCC700603) and *Enterobacter cloacae* (ATCC700323) were purchased from the National Center for Clinical Laboratories (National Clinical Laboratory Center of the Ministry of Health, China). A hypervirulent KP strain (HV) carrying the *rmpA* gene was kindly provided by the Affiliated Hospital of Ningxia University (Yinchuan, China). Bacteria were grown in solid culture on Columbia blood agar medium (Antu Biotechnology Engineering Co., Ltd., Zhengzhou, China) and in liquid culture in cation-adjusted Mueller–Hinton broth (CAMHB; Haibo Bio, Qingdao, China).

### Screening for tigecycline-insensitive strains

The minimum inhibitory concentrations (MICs) of tigecycline against various MDR strains were determined with the broth double microdilution method (9, 10). The bacterial suspensions were adjusted to the 0.5 McFarland standard (1 × 10⁸ CFU/mL) and diluted 1:20. The concentrations of tigecycline (GAR-936; MedChemExpress Life Science Reagents) ranged from 64 to 0.0065 µg/mL. Susceptibility was interpreted according to the Clinical and Laboratory Standards Institute (CLSI) guidelines (11). As a drug sensitivity breakpoint standard for tigecycline is not recommended by the CLSI, the interpretation was made with reference to the dilution method breakpoints for Enterobacteriaceae bacteria recommended by the US Food and Drug Administration (12): susceptible (S) ≤ 2 µg/mL, intermediate (I) = 4 µg/mL, and resistant (R) ≥ 8 µg/mL. *Klebsiella pneumoniae* (ATCC700603) and *Enterobacter cloacae* (ATCC700323) were used as the QC strain.

### Whole-genome sequencing and bioinformatic analysis

Genomic DNA was extracted with a TIANamp Bacteria DNA Kit (Tiangen Biochemical Technology Co., Ltd., Beijing, China). The extracted DNA was sequenced on the Illumina NovaSeq 6000 and PacBio platforms. The raw data files were analyzed and processed with CASAVA (Illumina, San Diego, CA, USA) for base calling. Before assembly, the raw reads were filtered to remove reads with >40% bases having Phred quality scores ≤20, reads containing >10% ambiguous bases (N), and reads with adapter contamination (overlap > 15 bp, mismatches < 3). The genome was assembled with the following steps: (i) the initial drafts of all 32 TIKP strains were assembled into a framework diagram with SPAdes (v4.2.0); (ii) miniasm and Racon (v1.08) were used to add the third-generation data to the framework diagram, and bridges were created using the long-read third-generation data; (iii) any conflicts were resolved with Unicycler (https://github.com/rrwick/Unicycler), and high-score bridges were selected on the basis of the quality score corresponding to the created bridge; (iv) GapCloser software (http://sourceforge.net/projects/soapdenovo2/files/GapCloser/) was used to fill the rest of the local internal spaces, and SOAPdenovo was used to finally assemble the genomes (13). Resistance genes were identified by comparing the predicted gene sequences with the Comprehensive Antibiotic Resistance Database (CARD; https://card.mcmaster.ca/) using TBLASTN (14). Virulence genes were identified by querying the predicted gene sequences with the Virulence Factor Database (VFDB; setA_pro, http://www.mgc.ac.cn/VFs/) using TBLASTN (15). Plasmid replicon types were identified with a BLASTN comparison against the PlasmidFinder (v2.0) Enterobacteriaceae plasmid database (16). Sequence types (STs) were determined with the MLST 2.0 web tool (https://cge.food.dtu.dk/services/MLST/) provided by the Center for Genomic Epidemiology (17). GrapeTree was used to visualize the ST relationships. SNPs were identified with SAMtools (v1.17). Low-quality SNPs (read depth < 5) and SNPs within 5 bp of another on the same chromosome were removed (18). A phylogenetic tree based on the core genome SNPs was constructed with FastTree v2.1.10 and the maximum-likelihood method.

### String test

A sterile loop was used to gently lift each colony vertically, on which it was inoculated with Columbia blood agar. Mucus filaments that formed both times the lifting step were repeated, and those that were >5 mm indicated a positive hypermucous phenotype (19), and that strain was deemed a hypermucous strain.

### Biofilm formation assay

A logarithmic-phase bacterial suspension was prepared with CAMHB broth to a turbidity of 0.5 McFarland standard, diluted 1:10, and added to the wells of a 96-well plate. Sterile CAMHB (negative control) and the hypervirulent strain HV (positive control) were used as the control wells, and all the plates were incubated statically at 37°C for 24 h. Planktonic cells were removed by washing the plates with saline solution. After the plates were air-dried completely at room temperature, the biofilms were fixed with 200 µL of methanol, air-dried, stained with 200 µL of crystal violet for 20 min, washed three times with deionized water, and air-dried again. Absolute ethanol (200 µL) was added to each well, and the samples were incubated in the dark for 10 min to allow the dissolution of the crystal violet. The samples were then transferred to a new sterile 96-well plate to measure the optical density at 600 nm (OD_600_). All the assays were performed in triplicate. For the judgment criterion, cutoff A was the mean OD₆₀₀ of the negative control. Strains were classified as a non-biofilm former (measured OD_600_ < A), weak biofilm former (A ≤ measured OD₆₀₀ < 2A), or strong biofilm former (measured OD_600_ ≥ 2A) (9).

### *Galleria mellonella* infection assay

Larvae (200–400 mg) with similar vitality and responses to stimuli were selected (*n* = 10 per group). Larvae injected with the hypervirulent HV strain constituted the positive control; the negative control larvae were injected with saline. Bacterial suspensions (1 × 10⁹ and 1 × 10⁸ CFU/mL) were prepared. A sample (10 µL) of suspension was injected into the posterior right ventral foot of each larva with a syringe (30G, 60 mm). The larvae were incubated at 37°C in sterile Petri dishes and monitored for mortality every 12 h. The criteria for insect death were no response to external stimuli or the insect becoming black. The dead larvae of each group were infected with 1 × 10⁹ CFU/mL for 12 h, collected, immersed in a 4% paraformaldehyde solution for 24 h, and embedded in paraffin. The paraffin sections were immersed in sequence in Environmentally Friendly Dewaxing Transparent Liquid I or II (G1128-1 L; Servicebio, China) for 20 min. The sections were placed into hematoxylin solution for 3–5 min and rinsed with tap water, after which they were placed in 95% ethanol for 1 min and stained with eosin for 15 s (H&E, G1076, Servicebio, China). Gram staining was used to determine the degree of bacterial infection and tissue damage during pathological examination (20).

### Serum resistance assay

A logarithmic-phase bacterial suspension was adjusted to 1 × 10^5^ or 1 × 10^4^ CFU/mL. Samples (25 µL) of the suspensions were mixed with 75 µL of pooled human serum and vortexed (ethics code: 2025-388). Aliquots were removed after 0, 60, 120, and 180 min, serially diluted 10-fold, plated on tryptic soy agar, and incubated at 37°C for 24 h. The colonies formed were counted (CFU/mL). The assays were performed in triplicate. Serum resistance levels were categorized as 1–6 on the basis of the colony survival rate (21): levels 1–4 = serum sensitive (1–2: highly sensitive; 3–4: moderately sensitive); levels 5–6 = serum resistant. The colony survival rate was designated B. The criteria used for classification were as follows: level 1: B < 10% at 60 or 120 min, <0.1% at 180 min; level 2: B = 10%–100% at 60 min, <10% at 180 min; level 3: B > 100% at 60 min, <100% at 120 or 180 min; level 4: B > 100% at 60 or 120 min, <100% at 180 min; level 5: B > 100% at 60 and 120 min, relatively reduced at 180 min; level 6: B > 100% at 60, 120, and 180 min, after which the bacterial numbers increased progressively. The formula for determining the colony survival rate was as follows: (the number of colonies at each time point/the number of colonies of this strain at 0 min) × 100.

### Statistical analysis

Statistical analysis and graphing were performed with GraphPad Prism 8 and 10 software. The standard error of the mean (SEM) was calculated for normally distributed data. Measurement data were analyzed with one-way analysis of variance, and Dunnett’s method was used to compare the experimental and control groups. The Student–Newman–Keuls method was used to evaluate the differences within groups. *P* values < 0.05 were considered significant.

## RESULTS

### Antimicrobial susceptibility profiles

Thirty-two TIKP strains (21%) were identified among the 152 MDR-KP isolates tested, with tigecycline MICs ranging from 4 to 32 µg/mL. In the sensitivity test of tigecycline, 12 strains were classified as intermediate, and 20 strains were determined to be tigecycline resistant (Table 1). The 32 TIKP isolates were predominantly resistant to the compounds Xinnuoming, cefuroxime axetil, cefuroxime, cefoxitin, and levofloxacin. Xinnuoming-resistant strains accounted for 62.5% of the strains. Only one strain was sensitive to cefuroxime axetil and cefuroxime, while the others were intermediate or resistant; these TIKP strains were resistant to levofloxacin. Cefoxitin-intermediate and resistant strains accounted for 68.8% of the strains. The MICs of compound Xinnuoming exceeded 320 µg/mL, and those of cefuroxime axetil, cefuroxime, and cefoxitin exceeded 64 µg/mL for some isolates (Table 1). Fourteen isolates were ESBL positive (Table 1). The results for the QC strain of *Klebsiella pneumoniae* (ATCC700603) and *Enterobacter cloacae* (ATCC700323) were within acceptable ranges, indicating that the proportion of TIKP isolates with a multidrug resistance phenotype was relatively high.

**TABLE 1:** Antimicrobial susceptibility results for 32 tigecycline non-sensitive *Klebsiella pneumoniae* strains^*a*^^,*b*^

Antibiotics	Strains																															
	2	3	4	6	7	8	9	10	12	13	14	15	16	17	18	19	20	21	22	23	24	26	27	28	29	30	31	32	34	35	37	38
Tigecycline	8/R	4/I	8/R	4/I	16/R	16/R	8/R	16/R	16/R	16/R	16/R	4/I	16/R	8/R	4/I	4/I	8/I	16/R	8/R	16/R	4/I	4/I	16/R	16/R	4/I	4/I	16/R	4/I	4/I	8/R	16/R	32/R
Compound Xinnuoming	≥320/R	≥320/R	≥320/R	≥320/R	≥320/R	≥320/R	≥320/R	≥320/R	2/S	2/S	≥320/R	2/S	2/S	2/S	2/S	2/S	≥320/R	2/S	80/R	2/S	≥320/R	2/S	≥320/R	≥320/R	80/R	2/S	2/S	≥320/R	160/R	≥320/R	≥320/R	≥320/R
Cefuroxime axetil	≥64/R	≥64/R	≥64/R	16/I	≥64/R	≥64/R	32/R	≥64/R	≥64/R	32/R	≥64/R	16/I	32/R	32/R	16/I	8/I	16/I	16/I	32/R	32/R	≥64/R	16/I	≥64/R	2/S	≥64/R	16/I	≥64/R	32/R	≥64/R	≥64/R	32/R	32/R
Cefuroxime	≥64/R	≥64/R	≥64/R	16/I	≥64/R	≥64/R	32/R	≥64/R	≥64/R	32/R	≥64/R	16/I	32/R	32/R	16/I	2/S	16/I	16/I	32/R	32/R	≥64/R	16/I	≥64/R	16/I	≥64/R	16/I	≥64/R	32/R	≥64/R	≥64/R	32/R	32/R
Cefoxitin	≥64/R	2/S	≥64/R	2/S	2/S	2/S	2/S	≥64/R	≥64/R	≥64/R	32/R	16/I	≥64/R	≥64/R	16/I	2/S	2/S	16/I	≥64/R	≥64/R	≥64/R	32/R	≥64/R	16/I	≥64/R	16/I	≥64/R	32/R	16/I	32/R	16/I	≥64/R
Levofloxacin	≥8/R	≥8/R	≥8/R	≥8/R	4/R	≥8/R	≥8/R	≥8/R	≥8/R	1/I	1/I	1/I	≥8/R	1/I	4/R	≥8/R	≥8/R	4/R	2/R	1/I	≥8/R	1/I	≥8/R	≥8/R	≥8/R	4/R	≥8/R	≥8/R	≥8/R	≥8/R	4/R	≥8/R
Ceftriaxone	≥32/R	≥64/R	≥64/R	2/S	≥64/R	≥64/R	32/R	≥64/R	≥64/R	2/S	≥64/R	8/R	2/S	2/S	2/S	2/S	2/S	2/S	2/S	2/S	≥64/R	2/S	≥64/R	32/R	8/R	2/S	2/S	2/S	≥64/R	≥64/R	2/S	2/S
Amoxicillin/clavulanic acid	≥32/R	16/I	≥32/R	2/S	2/S	≥32/R	2/S	≥32/R	2/S	2/S	16/I	2/S	2/S	2/S	2/S	2/S	2/S	2/S	2/S	2/S	≥32/R	2/S	≥32/R	2/S	≥32/R	2/S	≥32/R	≥32/R	16/I	2/S	≥32/R	2/S
Piperacillin/tazobactam	≥128/R	32/R	≥128/R	2/S	32/R	≥128/R	32/R	2/S	2/S	2/S	64/R	2/S	2/S	2/S	2/S	2/S	2/S	2/S	2/S	2/S	≥128/R	2/S	≥128/R	32/R	≥128/R	2/S	2/S	≥128/R	≥128/R	2/S	≥128/R	2/S
Ceftazidime	2/S	32/R	≥64/R	2/S	32/R	32/R	16/R	32/R	2/S	2/S	8/I	2/S	2/S	2/S	2/S	2/S	2/S	2/S	2/S	2/S	32/R	2/S	≥64/R	8/I	32/R	2/S	32/R	2/S	≥64/R	32/R	2/S	2/S
Cefepime	2/S	≥32/R	≥64/R	2/S	≥32/R	≥32/R	2/S	≥32/R	≥32/R	2/S	≥32/R	2/S	2/S	2/S	2/S	2/S	2/S	2/S	2/S	2/S	≥32/R	2/S	≥32/R	2/S	2/S	2/S	2/S	2/S	≥32/R	2/S	2/S	2/S
Cefoperazone/sulbactam	2/S	≥64/R	≥64/R	2/S	≥64/R	≥64/R	2/S	2/S	2/S	2/S	≥64/R	2/S	2/S	2/S	2/S	2/S	2/S	2/S	2/S	2/S	≥64/R	2/S	≥64/R	2/S	≥64/R	2/S	≥64/R	2/S	32/I	2/S	2/S	2/S
Ertapenem	2/S	2/S	≥8/R	2/S	2/S	2/S	2/S	2/S	2/S	2/S	2/S	2/S	2/S	2/S	2/S	2/S	2/S	2/S	2/S	2/S	2/S	2/S	≥8/R	2/S	2/S	2/S	2/S	2/S	2/S	2/S	2/S	2/S
Imipenem	2/S	2/S	≥16/R	2/S	2/S	2/S	2/S	2/S	2/S	2/S	2/S	2/S	2/S	2/S	2/S	2/S	2/S	2/S	2/S	2/S	2/S	2/S	≥16/R	2/S	2/S	2/S	2/S	2/S	2/S	2/S	2/S	2/S
Amikacin	2/S	2/S	≥16/R	2/S	2/S	2/S	2/S	≥64/R	2/S	2/S	2/S	2/S	2/S	2/S	2/S	2/S	2/S	2/S	2/S	2/S	2/S	2/S	≥64/R	2/S	2/S	2/S	2/S	2/S	2/S	2/S	2/S	2/S
Nitrofurantoin	8/R	N/A	N/A	N/A	N/A	N/A	6/R	N/A	N/A	N/A	N/A	N/A	N/A	N/A	N/A	6/R	N/A	14/R	N/A	N/A	9/R	N/A	6/R	7/R	N/A	N/A	1/S	12/R	10/R	N/A	N/A	N/A
ESBL	P	P	N	N	P	P	P	P	P	N	P	N	N	N	N	N	N	N	N	N	P	N	N	P	P	N	N	P	P	P	N	N

^*a*^ The interpretation criteria for the susceptibility breakpoints of tigecycline are as follows: sensitivity (S) ≤ 2 mg/L, intermediate (I) = 4 mg/L, and resistance (R) ≥ 8 mg/L.

^*b*^ N/A, not applicable; P, positive; N, negative.

### Clinical characteristics of the TIKP isolates

The 32 TIKP isolates originated primarily from the respiratory medicine department (28.1%) and intensive care unit (ICU; 15.6%) of the hospital. The main sample sources were sputum (46.9%) and urine (28.1%). Other sources included secretions (9.4%), whole blood (6.3%), drains (3.1%), and chest fluid and ascites (3.1%). The main types of infection included pneumonia (56.3%), urinary tract infection (18.8%), and abdominal infection (3.1%). Other types included soft-tissue infection, fever, bronchitis combined with pyelonephritis, nephrapostasis, ileus, hypostasis, and hepatapostema, each represented by a single case. Furthermore, in terms of age, elderly people (>60 years old) accounted for the majority (59.4%), and the others were young people (<50 years old) (18.8%) and middle-aged people (50–60 years old) (21.9%); the gender ratio was exactly half and half. Seventeen of the 32 infected patients (53.1%) had underlying disease, and three patients died (9.4%) (Table 2).

**TABLE 2:** Summary of clinical information for 32 tigecycline non-sensitive *Klebsiella pneumoniae* strains^*a*^

Isolates	Age	Sex	Specimen	Ward	Infection	Primary disease	Antibiotics	Prognosis
2	80	F	Urine	Older medicine	Urinary tract infection	Cerebral infarction, hypertension, heart failure	LEV	Cure
3	77	F	Phlegm	Dept. of Respiration	Pneumonia	Hypertension	SCF	Recovery
4	60	M	Phlegm	Dept. of Respiration	Pneumonia	N	ETP, ERT	Recovery
6	47	M	Phlegm	Dept. of Respiration	Pneumonia	Diabetes	SCF	Recovery
7	74	M	Phlegm	Rehabilitation Dept	Pneumonia	Hypertension, cerebral infarction	MEM	Discharged
8	72	M	Chest and ascites	ICU Dept	Abdominal infection	N	CRO, CEF	Recovery
9	52	F	Urine	Rehabilitation Dept	Urinary tract infection	Hypertension	Nitrofurantoin	Recovery
10	76	F	Phlegm	Dept. of Respiration	Pneumonia	N	MEM, P/T, ETP	Recovery
12	80	F	phlegm	Dept. of Respiration	Pneumonia	N	SCF, P/T, LEV	Death
13	78	M	Phlegm	Older medicine	Pneumonia	Hypertension	CAZ, LEV	Recovery
14	78	F	Drains	ICU Dept	Pneumonia	Hypertension, diabetes	SCF, CRO	Death
15	48	F	Secretion	Endocrinology Dept	Skin soft-tissue infection	N	P/T	Recovery
16	53	M	Phlegm	Cardio-thoracic Surgery Dept	Pneumonia	N	LEV	Recovery
17	72	M	Phlegm	General Practice Department	Pneumonia	N	SCF	Recovery
18	79	M	Phlegm	Dept. of Respiration	Pneumonia	Diabetes	P/T	Recovery
19	75	F	Urine	Cardiovasology Dept	Fever	Diabetes		Cure
20	80	M	Phlegm	ICU Dept	Pneumonia	N	IPM, TGC	Death
21	52	F	Urine	Dept. of Respiration	Bronchitis combined with pyelonephritis	Hypertension, diabetes	ETP	Cure
22	67	F	Phlegm	Neurosurgery Dept	Pneumonia	Hypertension	P/T	Discharged
23	53	F	Secretion	Urology Surgery Dept	Nephrapostasis	N	CRO	Recovery
24	74	F	Urine	Dept. of Respiration	Pneumonia	Hypertension	SCF	Recovery
26	65	M	Phlegm	ICU Dept	Pneumonia	N	PG, CXM	Recovery
27	48	F	Urine	Neurosurgery Dept	Urinary tract infection	Hypertension	PHM	Recovery
28	52	F	Urine	Rehabilitation Dept	Urinary tract infection	Hypertension	Nitrofurantoin	Recovery
29	82	M	Whole blood	General Surgery Dept	Pneumonia	Coronary heart disease	SCF, IPM, VA	Recovery
30	39	M	Phlegm	Cardio-thoracic Surgery Dept	Pneumothorax	N	LEV, CRO	Recovery
31	79	M	Urine	Urology Surgery Dept	Urinary tract infection	N	N	Recovery
32	79	F	Urine	ICU Dept	Ileus	Hypertension, cerebral infarction	PG, P/T	Recovery
34	39	M	Urine	Dept. of Gastroenterology	Urinary tract infection	N	MEM	Recovery
35	43	F	Whole blood	Obstetrics and Gynecology Dept	–	–	–	–
37	55	M	Phlegm	Neurosurgery Dept	Hypostatic pneumonia	N	P/T, CRO, LEV	Recovery
38	80	M	Secretion	General Surgery Dept	Hepatapostema	N	P/T, CRO	Recovery

^*a*^ N indicates no underlying diseases; – indicates missing data.

### Virulence and resistance gene profiles of 32 TIKP isolates

**Fig 1:**
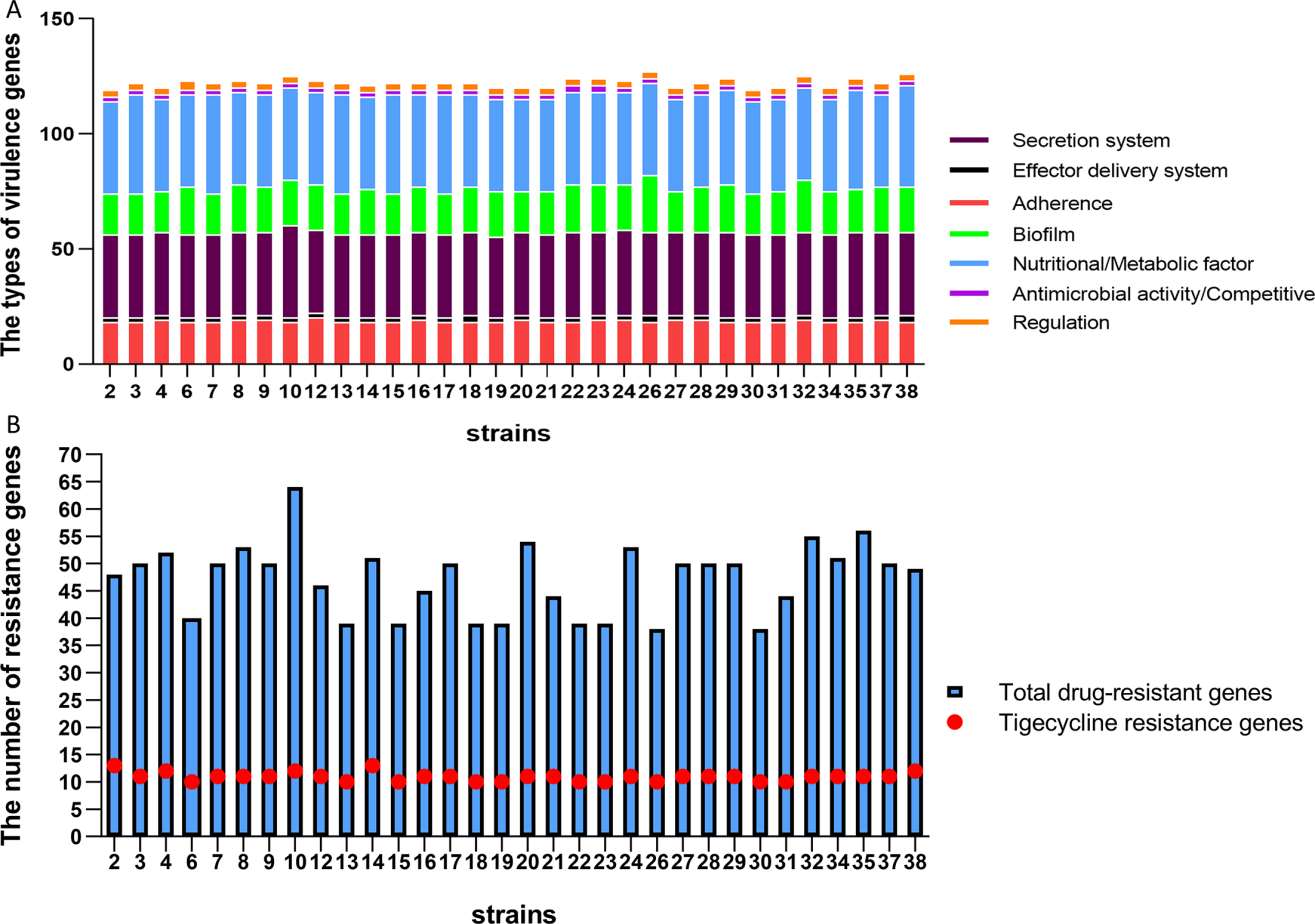
Types of virulence genes and number of resistance genes in 32 tigecycline-insensitive *Klebsiella pneumoniae* (TIKP) isolates. (**A**) Types of virulence genes. Different colors represent the classification of virulence genes. (**B**) Number of resistance genes. The heights of the columns represent the total number of resistance genes, and the positions of the red dots indicate the number of tigecycline resistance genes (identity > 90%).

Before genome sequencing of the 32 strains, mass spectrometry was used for strain identification to confirm the absence of contamination. An N50 value greater than 0.15 Mbp (Table S1), clean Q20 and clean Q30 values greater than 90% were considered an acceptable quality threshold, with higher N50 values indicating better assembly continuity (Table S2). On the basis of the assembly results, an NCBI database BLAST comparison of 16S rDNA was conducted to identify the strains. According to the VFDB, the virulence factors carried by the 32 TIKP isolates were classified into seven major categories, totaling 119–127 virulence genes (Table S3): secretion systems (35–40 types), effector delivery systems (2–3 types), adherence (18–20 types), biofilm (18–25 types), nutritional/metabolic factors (40–44 types), antimicrobial activity/competitive advantage (2–3 types), and regulation (3–4 types) (Fig. 1A). Analysis using CARD revealed 38–63 resistance genes in the 32 TIKP isolates (Table S4), with strain 10 containing the most resistance genes (63 types). The 32 TIKP isolates encoded 10–13 tigecycline resistance-associated genes (Fig. 1B). In summary, the total number of virulence genes carried by each strain did not vary greatly, and the differences in the numbers of various gene types were also not significant among the 32 TIKP isolates. However, the types and quantities of resistance genes vary greatly.

### Sequence typing and plasmid typing of 32 TIKP isolates

**Fig 2:**
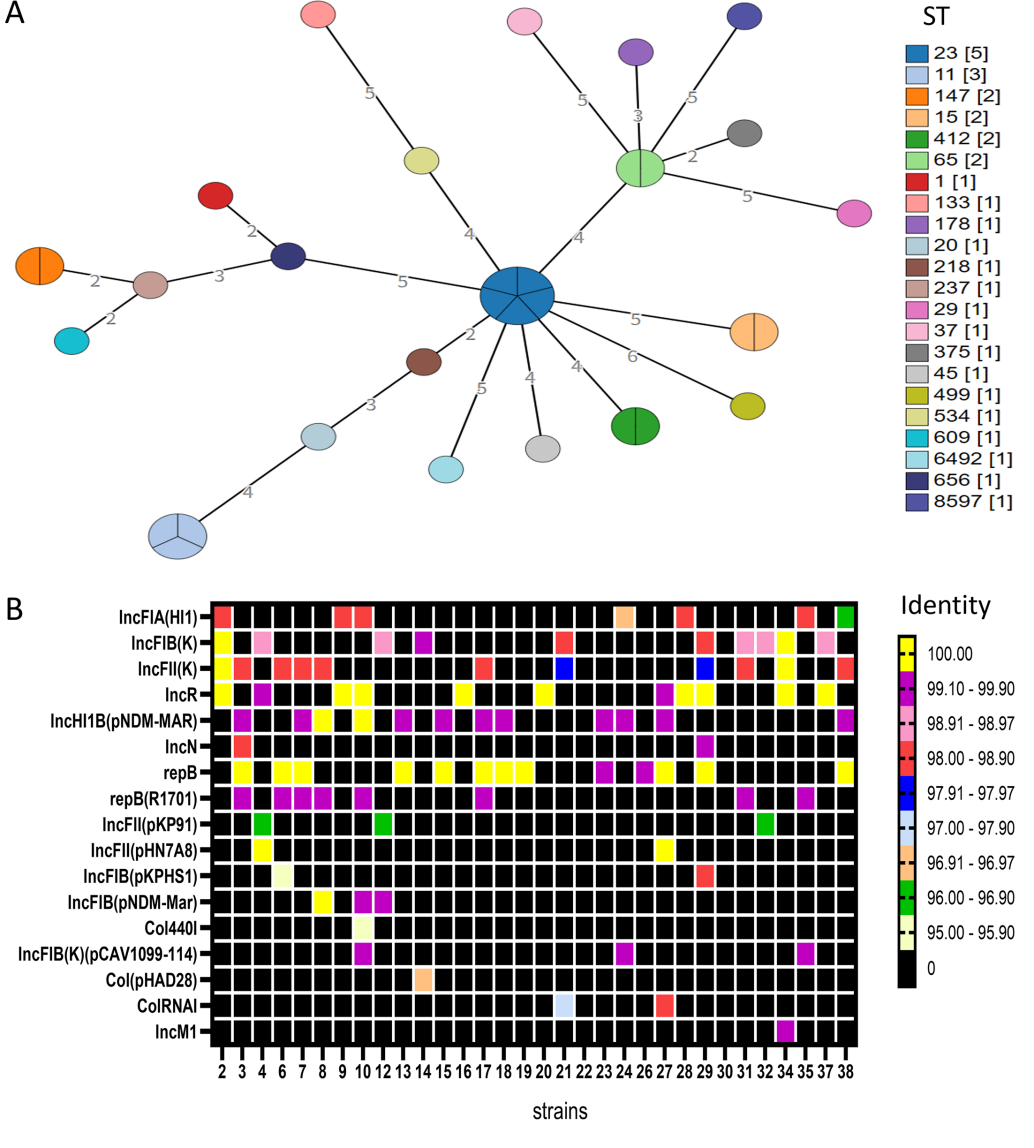
Sequence types (STs) and plasmid classification for 32 TIKP isolates. (**A**) ST classification. Each sphere or node represents a unique ST. The size of the node is related to the number of strains in each ST; i.e., the more strains, the larger the node. The color indicates the sample type. (**B**) Plasmid classification heatmap. Black represents no corresponding plasmid type, and the other colors represent the credibility of the different plasmid classifications (identity > 90%).

The McNally seven gene scheme, which includes *aarF*, *dfp*, *galR*, *glnS*, *hemA*, *rfaE*, and *speA*, was used for sequence typing (STs were assigned with MLST 2.0). MLST analysis revealed that the 32 TIKP strains included 21 ST subtypes. ST23 was most prevalent (five isolates: 3, 7, 13, 15, and 17), followed by ST11 (three isolates: 4, 27, and 35). The other STs were relatively diverse, and two strains each were assigned the following types: ST147 (strains 9 and 28), ST15 (strains 2 and 10), ST412 (strains 6 and 19), and ST65 (strains 18 and 38). One strain each was assigned the following types: ST1 (strain 31), ST133 (strain 29), ST178 (strain 14), ST20 (strain 22), ST218 (strain 26), ST237 (strain 8), ST29 (strain 32), ST37 (strain 12), ST375 (strain 30), ST45 (strain 21), ST499 (strain 16), ST534 (strain 37), ST609 (strain 24), ST656 (strain 34), and ST6492 (strain 23). Even more importantly, a novel allele (*phoE* 813) and a novel ST (8597; identified at https://bigsdb.pasteur.fr/) were identified for strain 20 (Fig. 2A). Overall, the ST distribution of the 32 TIKP isolates was somewhat scattered, and new alleles and new ST types were also observed.

The PlasmidFinder-2.0 server revealed that 30 isolates contained plasmids (excluding strains 22 and 30). Strain 10 contained the most plasmids (seven types). The predominant plasmid types were IncFIB(K) (10/32), IncFII(K) (11/32), IncR (11/32), RepB (13/32), and IncHI1B(pNDM-MAR) (12/32). Nine isolates contained both the RepB and IncHI1B (pNDM-MAR) plasmids (Fig. 2B). These plasmids represent the high-virulence evolution of hvKP and the phenotypes of β-lactamase carbapenem, β-cephalosporin, tigecycline, and MAR multidrug resistance, respectively.

### Phylogenetic analysis of 32 TIKP isolates

**Fig 3:**
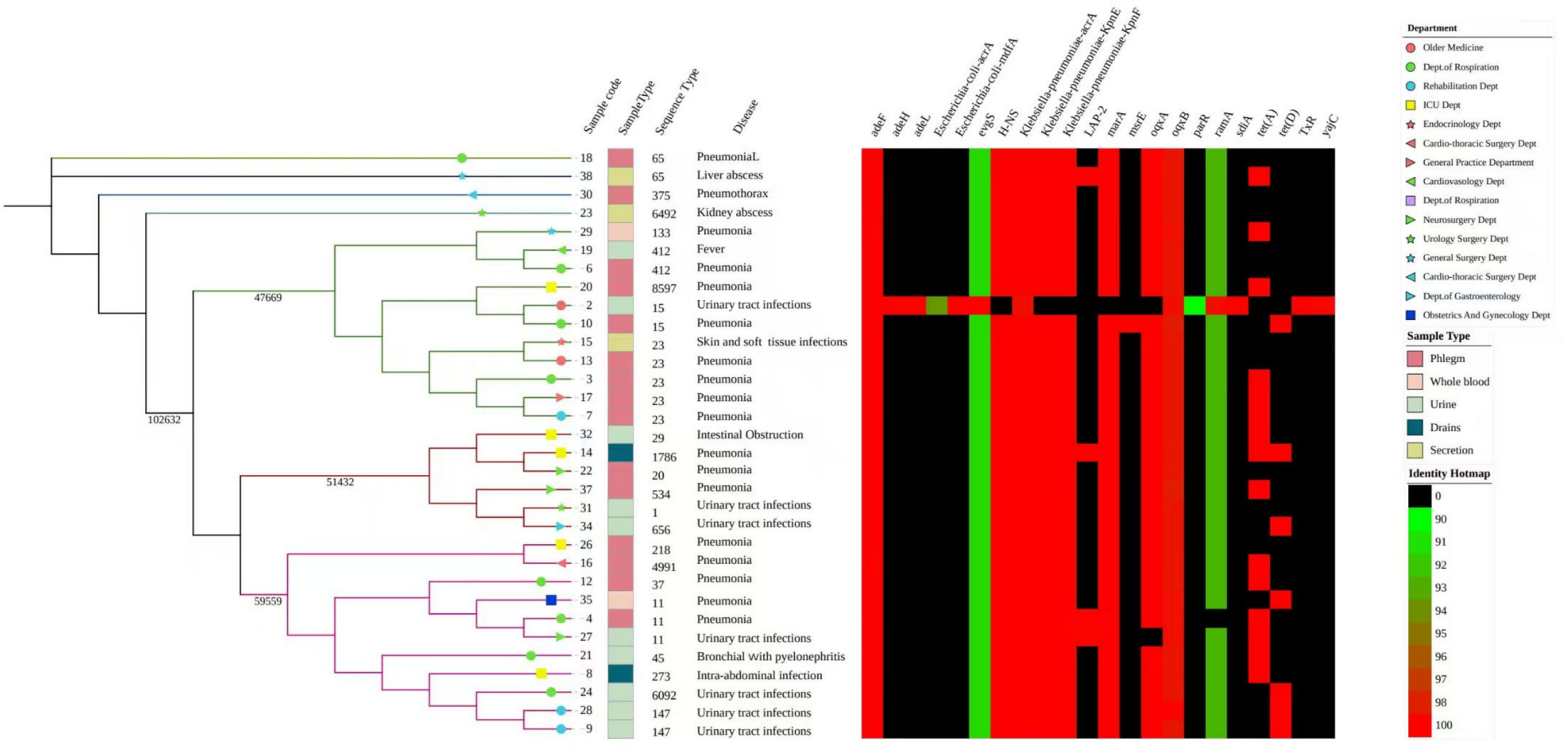
Phylogenetic tree of 32 TIKP isolates. From bottom to top, pink represents clade 1, red represents clade 2, and green represents clade 3. The numbers on the branches indicate the number of SNPs. Among the tigecycline resistance genes, black represents the absence of the corresponding gene, whereas red and green represent gene credibility (identity > 90%).

The genomic evolutionary relationships of the 32 TIKP isolates were compared in terms of the hospital department in which they were isolated, specimen type, disease, ST type, and tigecycline resistance gene profile (Fig. 3). The 32 isolates clustered into three main clades. Members of clade 1 (isolates 26, 16, 12, 35, 4, 27, 21, 8, 24, 28, and 9) were primarily from urine and whole blood and were associated with urinary tract infections (UTIs) and pneumonia. Members of clade 2 (isolates 32, 14, 22, 37, 31, and 34) were primarily from sputum and urine and were associated with UTIs and pneumonia. Members of clade 3 (isolates 29, 19, 6, 20, 2, 10, 15, 13, 3, 17, and 7) were primarily from whole blood and were predominantly associated with pneumonia. In total, clades 1–3 contained 102,632 core genomic SNPs. Although clades 1 and 3 each contained 11 isolates, they differed significantly in their SNP counts (clade 1: 47,669 SNPs; clade 3: 59,559 SNPs). Interestingly, although clade 2 was the smallest clade, it contained 51,432 SNPs. In general, the strains in clade 2 are significantly genetically distant and exhibit high genetic diversity. STs with similar sequences, such as ST23 and ST11, are more closely related. Although these 32 strains are from the same hospital in China, the relationships among them are relatively distant. Common tigecycline resistance genes (*adeF*, *evgS*, *kpnE*, *kpnF*, *marA*, *oqxA*, *oqxB*, *ramA*, and *tet*(A)) were widely detected but showed no clear evolutionary relationships.

### Resistance and virulence validation in highly virulent MDR TRKP strains

The isolates positive for the string test were strains 6, 7, **9**, 12, 13, 15, 18, 19, 21, 26, 27, 30, and **38**. Because strain 10 had the greatest number of plasmids and resistance genes, it was tested together with strains 9, 35, and 38 for biofilm formation, serum resistance, the *G. mellonella* assay, and targeted whole-genome sequencing to characterize the highly virulent MDR TRKP strains. We thus included both string-positive (9 and 38) and string-negative strains (10 and 35) and included all the tigecycline-resistant MICs (8, 16, and 32 µg/mL) for these strains in this analysis.

**Fig 4:**
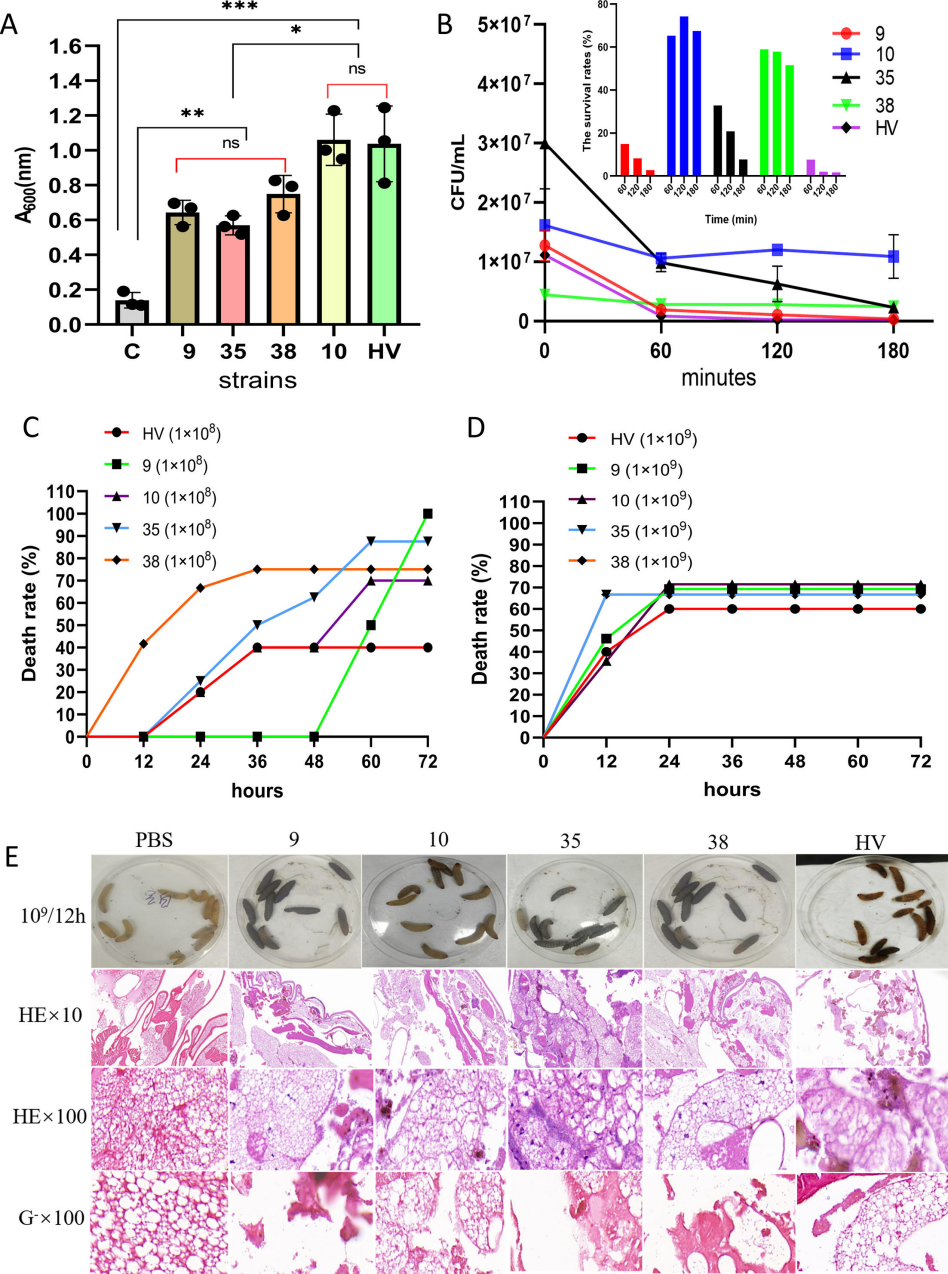
Verification of the resistance and virulence of highly virulent, multidrug-resistant TRKP strains. (**A**) Biofilm formation by TRKP strains. (**B**) Serum resistance of TRKP strains. (**C**) Invasive ability of TRKP strains (1 × 10^8^ CFU/mL) on *Galleria mellonella*. (**D**) Invasive ability of TRKP strains (1 × 10^9^ CFU/mL) on *G. mellonella*. (**E**) Larval state, hematoxylin–eosin and Gram staining. C is a blank control, and HV is a highly virulent control. Each experiment was repeated independently three times. *P ≤ 0.05; **P ≤ 0.01; ***P ≤ 0.001; ns, not significant.

The biofilm cutoff (A = OD_600_) was 0.140, and the OD_600_ values of the isolates ranged between 0.570 and 1.061. Therefore, all the isolates tested were strong biofilm formers. Compared with the control, isolates 9, 35, and 38 resulted in significantly greater biofilm formation (*P* < 0.01), with no significant differences among them (*P* > 0.05). Strain 10 and HA showed significantly greater biofilm formation than the control (*P* < 0.001), with no significant difference between the two. HV, a known hypervirulent KP strain, showed a similar capacity to form biofilm as that of strain 10. Notably, compared with strains 9, 35, or 38, strain 10 formed significantly more biofilm (*P* < 0.01) (Fig. 4A).

Serum resistance assays revealed that the survival rates of strain 9 at 60, 120, and 180 min were 14.9%, 8.24%, and 2.75%, respectively; those of strain 35 were 32.83%, 20.83%, and 7.67%, respectively; and those of strain HV were 7.62%, 2.02%, and 1.70%, respectively. Strains 9, 35, and HV all belonged to the level 2 sensitivity type. The survival rates of strain 10 at 60, 120, and 180 min were 65.32%, 74.3%, and 67.49%, respectively; those of strain 38 were 58.95%, 57.89%, and 51.58%, respectively. Strains 10 and 38 were also classified as serum sensitive but were between sensitivity levels 2 and 3. Strain 10 consistently displayed the highest survival rates at each time point, indicating its relatively greater serum resistance (Fig. 4B).

At a bacterial concentration of 1 × 10^8^ CFU/mL, strains 10, 35, and HV began to cause mortality in *G. mellonella* at 24 h. By 72 h, the mortality rates of these strains were 70%, 90%, and 40%, respectively. Strain 9 caused the death of the insects after 60 h, which reached 100% by 72 h. Strain 38 caused the death of the insects at 12 h, which reached 75% by 72 h (Fig. 4C). At a bacterial concentration of 1 × 10⁹ CFU/mL, death occurred by 12 h in all the groups. Mortality at 24–72 h after treatment with strain 9, 10, 35, or 38 remained at approximately 70%, but it was 60% with strain HV. Compared with strain HV, all the TRKP strains clearly caused higher final mortality at concentrations of 1 × 10⁹ CFU/mL, indicating that the four tigecycline-resistant strains were virulent strains (Fig. 4D). H&E staining of the dead larvae revealed disrupted internal structures in all the infected groups but intact structures in the saline control group. Gram staining confirmed the presence of gram-negative bacilli in the infected larvae (Fig. 4E).

### Genomic features of highly virulent MDR TRKP isolates

**Fig 5:**
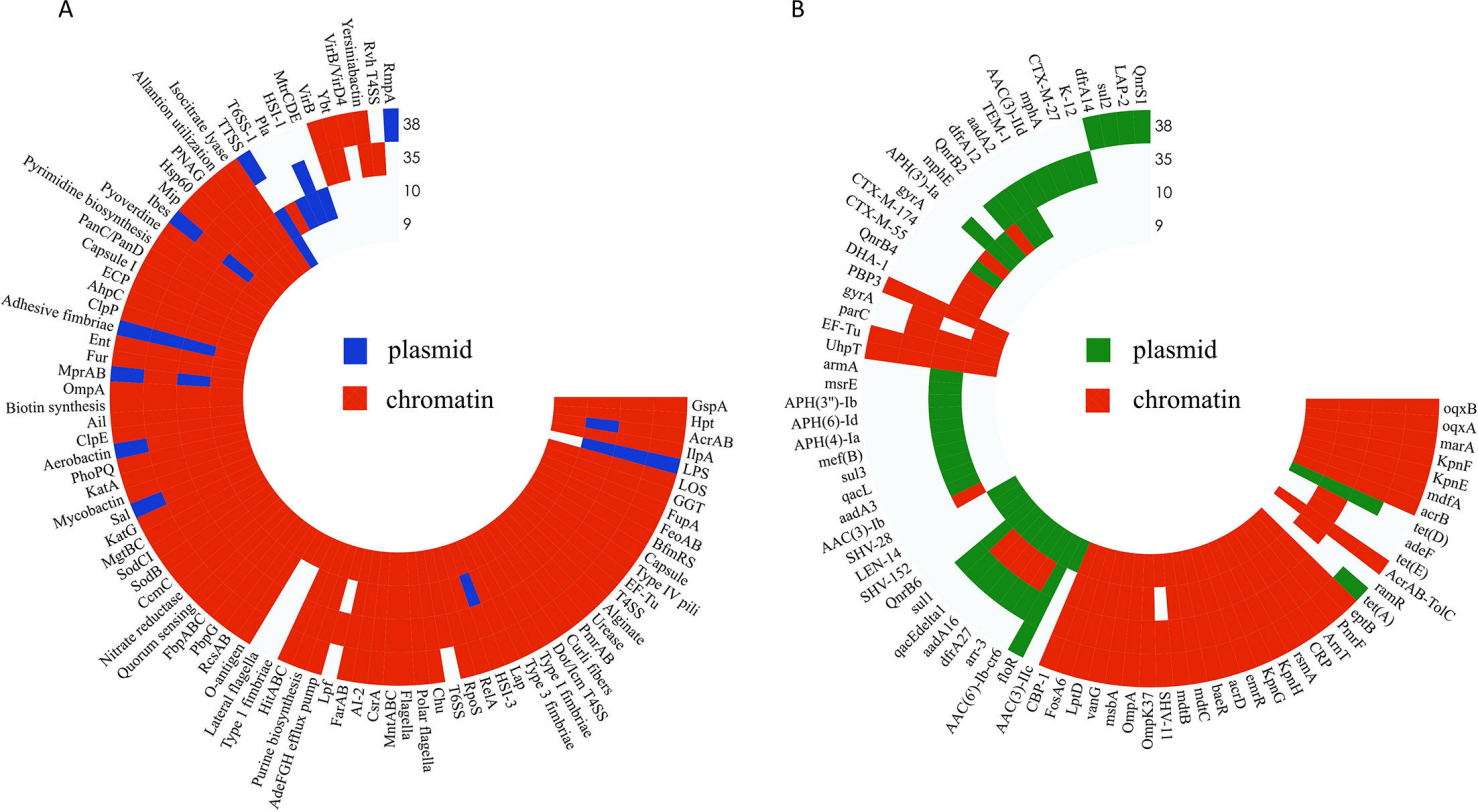
Alignments of the distributions of virulence and resistance genes. (**A**) Circle graphs of the distribution of virulence genes in TRKP strains. Red indicates that the virulence genes are located on the chromosome, blue indicates that they are located on a plasmid, and white indicates the absence of a corresponding virulence gene. (**B**) Circle graphs of the distribution of resistance genes in the TRKP strains. Red indicates that the resistance genes are located on the chromosome, green indicates that they are located on a plasmid, and white indicates the absence of a corresponding virulence gene (identity > 90%).

Comparison of the gene sequences of the MDR TRKP isolates with those of the VFDB revealed 76 virulence genes common to all four target strains (9, 10, 35, 38). The total number of virulence genes per strain was similar, supporting their high virulence potential (Fig. 5A). Eighty distinct resistance genes were identified across the four strains in the CARD database. Chromosomal resistance genes numbered 33 in strain 9, 45 in strain 10, 33 in strain 35, and 31 in strain 38. The number of plasmid-borne resistance genes was 12 in strain 9, 18 in strain 10, 17 in strain 35, and 6 in strain 38. Thirty resistance genes (*eptB*, *pmrF*, *arnT*, *crp*, *rsmA*, etc.) were present on the chromosomes of all four strains, with a 100% detection rate. The genes *aac(6′)-Ib-cr6*, *arr-3*, *dfrA27*, *aadA16*, and *qacE*delta1 were detected on both the chromosome and plasmids of strain 10. Strains 9, 10, 35, and 38 shared the tigecycline resistance genes *oqxB*, *oqxA*, *marA*, *kpnF*, *kpnE*, *mdfA*, and *acrB,* and those encoding the AcrAB-TolC efflux system. In addition, strain 10 contained *adeF*, *ramR*, *tet*(D), and *tet*(E) (Fig. 5B).

Strain 9 carried one plasmid encoding one virulence gene. Strain 10 carried five plasmids: 10-Plas1 (no resistance genes, six virulence genes), 10-Plas2 (18 resistance genes, four virulence genes), 10-Plas3 and 10-Plas4 (no resistance genes or virulence genes), and 10-Plas5 (17 resistance genes, two virulence genes, including tigecycline-resistance gene *tet*(D)). Strain 35 carried two plasmids: 35-Plas1 (seven resistance genes and three virulence genes) and 35-Plas2 (one virulence gene and 14 resistance genes, including the tigecycline resistance gene *tet*(D)). Strain 38 carried two plasmids: 38-Plas1 (six resistance genes and two virulence genes) and 38-Plas2 (six virulence genes) (Table 3). In summary, among the four strains, the resistance and virulence genes were colocalized on five plasmids (10-Plas2, 10-Plas5, 35-Plas1, 35-Plas2, and 38-Plas1).

**TABLE 3:** Resistant and virulent characterization plasmids of the TNSKP isolates^*a*^

Plasmids	Size (bp)	GC (%)	Resistance gene	Virulence genes	Integron/ISfinder	Cover/identity (%)	Description	Accession numbers
9Plas1-IncFII(K)	78,301	52.87	N	TTSS	AB616660/ISVsa	85.38/99.99	p20SC1-3AZ6BT	CP000648
10Plas1-IncFIB(K) (pCAV1099-114)	187,078	49.96	N	LPS, MprAB, Ibes, Pla, adhesive fimbriae, HSI-1	KF719970/ISSen3	97.16/99.97	p5921_tmexCD	CP011596
10Plas2-IncHI1B(pNDM-MAR)	278,257	46.78	AAC(3)-Ib, aadA3, qacL, sul3, mef(B), APH(4)-Ia, APH(6)-Id, APH(3′′)-Ib, APH(3′)-Ia, mphE, msrE, armA, sul1, DHA-1, QnrB4, APH(3′′)-Ib, APH(6)-Id, CTX-M-55	Ibes, MtrCDE, AcrAB, HSI-3	AY522431/ISEc9	94.99/99.96	pSXC4-2_tmex_350k	JN420336
10Plas3-no	4,439	44.97	N	N	PSEMT/N	100/99.23	pKqs_09A323_3	N
10Plas4-Col440I	4,574	54.89	N	N	PSEMT/N	99.98/100	pKP32558-7	CP023920
10Plas5-IncR	98,736	52.62	AAC(6′)-Ib-cr6, arr-3, dfrA27, aadA16, qacEdelta1, sul1, QnrB2, sul1, APH(3′)-Ia, rA12, aadA2, qacEdelta1, sul1, CTX-M-174, floR, tet(D)	TTSS, LPS	JX193301/ISPa38	82.17/100	p2019036D-mcr8-345kb	DQ449578
35Plas1-IncFIB(K) (pCAV1099-114)	177,256	51.36	APH(3′)-Ia, TEM-1, AAC(3)-IId, dfrA12, aadA2, qacEdelta1, sul1	HSI-1, LPS, adhesive fimbriae	JX101693/ISSen3	88.75/100	pHKU57_1	CP011596
35Plas2-repB (R1701)	77,484	51.54	QnrB2, sul1, CTX-M-27, floR, catII from *Escherichia coli* K-12, AAC(6′)-Ib-cr6, arr-3, dfrA27, aadA16, qacEdelta1, sul1, tet(D)	LPS	JX515588/ISPa38	84.05/99.96	p2019036D-mcr8-345kb	CP039970
38Plas1-IncFII(K)	120,943	53.34	dfrA14, sul2, floR, tet(A), LAP-2, QnrS1	Adhesive fimbriae, TTSS	KF719970/IS5075	99.92/99.99	pSW25tet_A	CP000648
38Plas2-repB	188,132	49.83	N	Sal, RmpA, LPS, Aerobactin, MprAB, Ibes	AY214164/ISYps3	99.73/99.99	p53322-228	AP006726

^*a*^ N indicates no.

## DISCUSSION

Tigecycline is often used as an antibiotic of last resort to combat MDR-KP. However, tigecycline resistance has been reported in KP with increasing frequency after treatment with tigecycline or even without exposure to tigecycline (22). We identified 32 TIKP strains (21%) among 152 MDR-KP isolates, which indicates that the rate of resistance to tigecycline is relatively high among MDR strains. Resistance to β-lactam antibiotics and aminoglycosides was the most frequently observed resistance phenotype. These TIKP strains were predominantly isolated from the respiratory medicine department and the ICU and mainly from sputum and urine samples, underscoring the widespread nature of drug-resistant KP infections.

Typical *Klebsiella pneumoniae* genomes are approximately 5–6 Mbp in size and encode about 5,000–6,000 genes. Among these, roughly 1,700 genes are conserved across all members of the species and are defined as core genes, whereas the remaining genes are variably present (23). Consistent with these reports, data from the NCBI database indicate that *K. pneumoniae* genome sizes range from approximately 5.0 to 6.5 Mbp. In the present study, the genomes of the analyzed isolates similarly varied in size, ranging from 5.18 to 6.50 Mbp. Notably, the types and numbers of resistance genes differed markedly among the 32 TIKP isolates, whereas the total number of virulence genes carried by each strain showed relatively little variation. Comparative genomic analysis yielded similar findings, demonstrating that although the antibiotic resistance profiles of 44 ST101 *K. pneumoniae* genomes are highly variable and associated with incompatible plasmid types, their virulence factor profiles remain largely conserved (24).

ST23 KP is a globally recognized hypervirulent clone that forms the predominant sequence type in CG23 (25). In China, ST23 is a major ST of hypervirulent strains, particularly in community-acquired infections, whose prevalence is especially high in southern regions (26). ST11 is a clone that is extremely common and dominant in China and has almost become synonymous with CRKP in China (27). In this study, ST23 was the most frequently observed ST among the TIKP isolates, followed by ST11, with various other STs, including the novel ST8597. A phylogenetic analysis revealed the closest genetic relationship between strains 28 and 9 (both belonging to ST147 and detected in the rehabilitation department). Strains 4 and 27 were also closely related and belonged to ST11. However, strain 35, which was also a member of ST11 and derived from the same ancestor, had a relatively more distant genetic relationship with these strains. Interestingly, the relationships between strains 16 and 26 were also relatively close (see graph), although their ST classifications differed. Similarly, the ST23 isolates (strains 3, 7, 13, 15, and 17) showed varying degrees of relatedness. This may be attributable to the high number of core genome SNPs (102,632) in these resistant isolates, which reflects the variability of the sequences among them.

IncFIB(K) plasmids are prevalent (>30%) in KP, particularly in ST23 and ST11 (28). Our data confirmed the co-occurrence of ST11 and IncFIB(K) in strains 4 and 27. The IncHI1B and RepB virulence plasmids often coexist (29). We observed that 75% (9/12) of IncHI1B (pNDM-MAR) plasmids were present with RepB (Fig. 2). The IncHI1B (pNDM-MAR) plasmid type was detected more frequently (12/32) in all TIKP strains because it carries New Delhi metal-β-lactamase (NDM)-heavy-metal-resistance genes (30) and can persist in hospital environments despite disinfection (31). At present, IncHI1B (pNDM-MAR) plasmids typically confer resistance to carbapenems, aminoglycosides, and sulfonamides, often leaving tigecycline as the only effective antibiotic (32). Alarmingly, all 12 IncHI1B (pNDM-MAR)-positive isolates in this study were tigecycline-insensitive, suggesting the potential prevalence of IncHI1B (pNDM-MAR) plasmids in Ordos, Inner Mongolia.

The EvgS/EvgA two-component system, which senses and responds to key signal transduction pathways under environmental stress (e.g., low pH, cations, antibiotics), activates efflux pumps, such as AcrAB-TolC, thus mediating tigecycline resistance (33). Other studies have shown that EvgS/EvgA may also phosphorylate EvgA via a four-step phosphorelay in response to environmental signals, increasing acid resistance and drug resistance in some hvKP strains (34). EvgS/EvgA can be found and conserved only in *Escherichia coli* and *Shigella* (35). However, our remarkably high *evgS* detection rate (96.88%) in TIKP may be specific to the Ordos region. Other highly prevalent genes (*acrA*, *ramA*, *marA*) are closely related to the expression of the external discharge pump AcrAB-TolC, and the transcription of *acrA* is regulated by RamA and MarA (36). Coregulation by RamA and MarA significantly enhances the efficiency of efflux compared with that regulated by single regulators (36, 37). Histone-like nucleoid-structuring protein (H-NS) may directly or indirectly promote biofilm formation of CRKP and lead to antibiotic resistance (38). The OqxAB efflux pump is composed of OqxA and OqxB and, when overexpressed, confers tigecycline resistance (39). In the present study, we detected the co-occurrence of H-NS and RamA/MarA in 93.72% (30/32) of the isolates, and the *OqxA* and *OqxB* genes were detected in almost 100% of the 32 strains of TIKP, except for strains 2 and 27, which may be an important reason for the increased rate of resistance to tigecycline in TIKP. The KpnEF efflux system is composed of KpnE and KpnF and contributes to tigecycline resistance by enhancing the ability of cells to pump out tigecycline (40). According to our results, KpnE and KpnF were present in all but two strains. Therefore, AcrAB-TolC, OqxAB, and KpnEF appear to be the primary efflux mechanisms in these 32 TIKP strains, although this requires further functional validation.

10-Plas1 is similar to the KP plasmid p5921_tmexCD (97.16% coverage), and 10-Plas2 is similar to the KP plasmid pSXC4-2_tmex_350k (94.99% coverage). Both plasmids carry the *tmexCD–toprJ* gene cluster, encoding an RND efflux pump, which confers high-level tigecycline resistance, and often also carry carbapenemase genes (41). However, 10-Plas1 contained only six virulence genes, whereas 10-Plas2 carried 18 resistance genes and four virulence genes. This discrepancy could be attributable to the continuous self-evolution of the strain in the presence of clinical polypharmacy and disinfectants. Moreover, the *Escherichia coli* insertion sequence ISEc9 and integron AY522431 were integrated into the 10-Plas2 plasmid (Table 3), suggesting that resistance “migrated” from *E. coli*.

10-Plas5 matches the KP plasmid p2019036D-mcr8-345kb (82.17% coverage), which carries the colistin resistance gene *mcr-8* and its variants and often coexists with *tmexCD–toprJ* on the same plasmid or in the same strain (42). The co-occurrence of *tmexCD1–toprJ1* and *mcr-8.5* confers dual resistance to tigecycline and colistin, severely limiting therapeutic options (43). Studies have also suggested that the *mcr-8/tmexCD1–toprJ1*-containing plasmid originated from animal isolates and enters clinical settings via the food chain (44). In the present study, strain 10 not only exhibited robust biofilm-forming capacity and serum resistance but also presented the greatest number of plasmid structures and antimicrobial resistance genes among the four strains analyzed. The concurrent presence of both *mcr-8* and *tmexCD–toprJ* in strain 10 indicates that the pattern of co-occurrence of these genes is established in this region of Inner Mongolia. Notably, strain 10 tested negative on the string test, demonstrating that this test solely reflects the virulence phenotype and is not indicative of the genomic complexity of a strain.

In summary, tigecycline is commonly used as a last-resort antibiotic for the treatment of multidrug-resistant *Klebsiella pneumoniae* (MDR-KP). In this study, we demonstrated that the proportion of tigecycline-insensitive *K. pneumoniae* (TIKP) strains was high among MDR-KP isolates, with most originating from the respiratory medicine department and the intensive care unit. Although all 32 isolates were collected from the same hospital in China, they exhibited substantial heterogeneity in resistance gene profiles, sequence types (STs), plasmid types, and genetic distances. One isolate showed strong biofilm-forming ability and the highest serum resistance. This strain carried the largest number of plasmids, and both its chromosome and plasmids harbored numerous resistance genes, including an increased number of tigecycline resistance determinants, as well as the concurrent presence of *mcr-8* and *tmexCD–toprJ*. Notably, this isolate did not exhibit a hypermucoviscous phenotype but instead displayed a more complex genomic architecture.

## Data Availability

The whole-genome sequence data were deposited at the NCBI Sequence Read Archive (SRA) under the BioProject accession number PRJNA1303952.

## SUPPLEMENTAL MATERIAL

Table S1 spectrum.03228-25-s0001.xlsx All sample scaffold statistics.

Table S2 spectrum.03228-25-s0002.xlsx All sample clean data statistics.

Table S3 spectrum.03228-25-s0003.xlsx The number of virulence genes in all samples.

Table S4 spectrum.03228-25-s0004.xlsx Detailed information on the resistance genes of all samples.

ASM does not own the copyrights to Supplemental Material that may be linked to, or accessed through, an article. The authors have granted ASM a non-exclusive, world-wide license to publish the Supplemental Material files. Please contact the corresponding author directly for reuse.
